# Decelerated epigenetic aging associated with mood stabilizers in the blood of patients with bipolar disorder

**DOI:** 10.1038/s41398-020-0813-y

**Published:** 2020-05-04

**Authors:** Satoshi Okazaki, Shusuke Numata, Ikuo Otsuka, Tadasu Horai, Makoto Kinoshita, Ichiro Sora, Tetsuro Ohmori, Akitoyo Hishimoto

**Affiliations:** 1 Department of Psychiatry, Kobe University Graduate School of Medicine, Kobe, Japan; 2 Department of Psychiatry, Graduate School of Biomedical Sciences, Tokushima University, Tokushima, Japan

**Keywords:** Clinical genetics, Bipolar disorder

## Abstract

There is high mortality among patients with bipolar disorder (BD). Studies have reported accelerated biological aging in patients with BD. Recently, Horvath and Hannum et al. independently developed DNA methylation (DNAm) profiles as “epigenetic clocks,” which are the most accurate biological age estimate. This led to the development of two accomplished measures of epigenetic age acceleration (EAA) using blood samples, namely, intrinsic and extrinsic EAA (IEAA and EEAA, respectively). IEAA, which is based on Horvath’s clock, is independent of blood cell counts and indicates cell-intrinsic aging. On the other hand, EEAA, which is based on Hannum’s clock, is associated with age-dependent changes in blood cell counts and indicates immune system aging. Further, Lu et al. developed the “GrimAge” clock, which can strongly predict the mortality risk, and DNAm-based telomere length (DNAmTL). We used a DNAm dataset from whole blood samples obtained from 30 patients with BD and 30 healthy controls. We investigated Horvath EAA, IEAA, Hannum EAA, EEAA, Grim EAA, DNAmTL, and DNAm-based blood cell composition. Compared with controls, there was a decrease in Horvath EAA and IEAA in patients with BD. Further, there was a significant decrease in Horvath EAA and IEAA in patients with BD taking medication combinations of mood stabilizers (including lithium carbonate, sodium valproate, and carbamazepine) than in those taking no medication/monotherapy. This study provides novel evidence indicating decelerated epigenetic aging associated with mood stabilizers in patients with BD.

## Introduction

Bipolar disorder (BD) is a recurrent chronic mental disorder characterized by periods of depressive and manic states that affects ~1% of the general population^1^. A high mortality rate has been observed in patients with BD, resulting from both natural causes (such as cardiovascular and cerebrovascular diseases) and unnatural causes (such as suicide and accident)^2^. The accelerated aging hypothesis of BD has been proposed to explain these observations. Many aging features have been reported in patients with BD^3,4^. An assessment of biological age depends on measuring relevant “biological clocks.” Telomere length (TL) is the most commonly used biological clock, although inconsistent results have been reported in BD. Several studies have shown accelerated telomere shortening in patients with BD^5–10^; however, other studies have observed no significant difference compared with controls^11,12^. Furthermore, others have demonstrated longer TL in patients with BD, resulting from the potential effects of lithium carbonate (Li), which is a first-line mood stabilizer^13,14^.

Aging research has progressed substantially in recent decades. One review concluded that epigenetic clocks were the most promising models among six potential biological clocks (epigenetic clocks, TL, predictors based on transcriptomics, proteomics, metabolomics, and composite biomarkers)^15^. Recently, Horvath and Hannum et al. independently established two broadly used epigenetic clocks based on DNA methylation (DNAm) profiles. Horvath’s clock was developed using 353 cytosine phosphate guanines (CpG) sites from multiple tissues from children and adults^16^. Hannum’s clock was developed using 71 CpG sites from blood samples from adults^17^. Horvath’s clock is independent of changes in blood cell composition, while Hannum’s clock is influenced by these changes^18^. Subsequently, Chen et al. developed intrinsic and extrinsic epigenetic age acceleration (IEAA and EEAA, respectively), which apply to blood samples only^19^. IEAA, which is based on Horvath’s clock, is independent of blood cell counts and indicates cell-intrinsic aging. On the other hand, EEAA, which is based on Hannum’s clock, is associated with age-dependent changes in blood cell counts and indicates immune system aging^20^. More recently, Lu et al. developed the DNAm GrimAge, which captures mortality and predicts lifespan and healthspan, using 1030 CpG sites from blood samples, based on a composite biomarker constructed from 10 clinical characteristics associated with “grim” news; namely, chronological age, sex, DNAm-based smoking pack-years, and seven DNAm-based estimators of plasma proteins^21^. One should adjust for age and sex before relating GrimAge to conditions, because it uses chronological age and sex in its definition. Furthermore, Lu et al. established an estimator for predicting DNAm-based TL (DNAmTL) based on 140 CpG sites from blood samples^22^.

Epigenetic age acceleration (EAA) measures are associated with diverse conditions, including Down syndrome^23^, Alzheimer’s disease^24^, Parkinson’s disease^25^, alcoholism^26^, insomnia^27^, higher self-discipline and lower economic strength^28^, exposure to violence in childhood^29^, summative lifetime stress^30^, completed suicide^31^, and all-cause mortality^32^. Studies using blood and postmortem brain samples have indicated accelerated epigenetic aging in major depressive disorder (MDD)^33,34^. A meta-analysis on posttraumatic stress disorder (PTSD) revealed accelerated epigenetic aging associated with childhood trauma and lifetime PTSD severity, although no association was observed between epigenetic aging and PTSD itself^35^. Our previous study reported decreased EEAA in patients with schizophrenia (SCZ)^36^; however, other studies using Horvath’s clock have reported no accelerated epigenetic aging in blood or postmortem brain samples from patients with SCZ^37–39^.

Studies have reported altered genome-wide DNAm profiles in both peripheral tissue and postmortem brain samples from patients with BD^40–42^. Moreover, three mood stabilizers (Li, sodium valproate [VPA], and carbamazepine [CBZ]) have been reported to commonly induce methylation of specific genes^43^. A previous study demonstrated a stronger correlation between DNAm age and chronological age in patients with BD with low suicidal behavior scores than in those with high scores^44^. Another study reported significantly accelerated epigenetic aging of blood in older, but not younger, patients with BD compared with non-psychiatric controls^45^. However, these studies only used Horvath’s clock and did not investigate the relationship between medications and epigenetic aging.

Previous studies have reported a common adaptability function between the affective and immunological systems^46^. Increased plasma levels of several inflammatory cytokines have been shown in patients with BD compared with those in healthy controls^47^. In addition, recent studies have demonstrated that patients with BD have a reduced total T cell percentage and cytotoxic CD8+ T cell population^48,49^, and that patients with BD type II have a lower CD4+ and CD8+ T cell percentage, which is possibly dependent on the current BD phase^50^.

In this study, we compared Horvath EAA, Hannum EAA, IEAA, EEAA, Grim EAA, age-adjusted DNAmTL, and DNAm-based blood cell composition between patients with BD and controls using a DNAm dataset of whole-blood samples. Furthermore, we assessed the effects of psychotropic medications including Li, VPA, and CBZ on epigenetic aging.

## Materials and methods

### Participants

This study was conducted in accordance with the Declaration of Helsinki and was approved by the institutional ethics committees of Kobe University Graduate School of Medicine and the University of Tokushima Graduate School. All the participants provided written informed consent after receiving a complete study description.

Our cohort consisted of 30 patients with BD and 30 healthy controls matched by age and sex, who were recruited from Tokushima University Hospital (Table 1). A diagnosis of BD was assessed according to the criteria of the Diagnostic and Statistical Manual of Mental Disorders, 4th Edition, Text Revision (DSM-IV-TR). Healthy controls were recruited from hospital staff, students, and company employees, who had no psychiatric problems or previous mental illness history^51^. All the participants were of Japanese descent and did not have any neurodevelopmental disorder, history of head injury or drug/alcohol abuse, and were not taking hormonal drugs.

**Table 1:** Demographic and clinical characteristics as well as measures of epigenetic age acceleration in our study.

	Control (*n* = 30)	Bipolar disorder (*n* = 30)	*P*-value
Bipolar type, I/II	–	20/10	
Sex, male/female	16/14	16/14	1.000^a^
Age (years), mean±SD	48.8 ± 11.3	49.0 ± 11.4	0.946^b^
Age of onset (years), mean±SD	–	34.7 ± 11.0	
Duration of illness (years), mean±SD	–	14.0 ± 11.7	
Medication, *n* (%), mean ± SD in users (mg/day)			
Medication-free	–	3 (10.0)	
Lithium carbonate	–	22 (73.3), 859 ± 220	
Sodium valproate	–	9 (30.0), 738 ± 298	
Carbamazepine	–	4 (13.3), 525 ± 150	
Lamotrigine	–	2 (6.7), 150 ± 71	
Antipsychotics (chlorpromazine equivalent)	–	15 (50.0), 236 ± 218	
Benzodiazepines (diazepam equivalent)	–	19 (63.3), 12.5 ± 9.7	
DNA methylation-based epigenetic age acceleration and age-adjusted telomere length			
Horvath EAA	1.166 ± 4.182	−1.166 ± 4.249	**0.0365**^b^
IEAA	1.116 ± 4.050	−1.116 ± 4.134	**0.0389**^b^
Hannum EAA	0.786 ± 3.914	−0.786 ± 4.047	0.132^b^
EEAA	1.015 ± 4.324	−1.015 ± 4.991	0.0975^b^
Grim EAA	0.169 ± 5.575	−0.169 ± 3.548	0.781^c^
DNAmTLAdjAge	−0.003 ± 0.165	0.003 ± 0.198	0.889^b^

*EAA* epigenetic age acceleration, *IEAA* intrinsic epigenetic age acceleration, *EEAA* extrinsic epigenetic age acceleration, *DNAmTLAdjAge* age-adjusted DNA methylation-based telomere length, *SD* standard deviation.

^a^*P*-value was calculated using Fisher’s exact test.

^b^*P*-value was calculated using Student’s *t*-test.

^c^*P*-value was calculated using Welch’s *t*-test.

Boldface type indicates significance.

### DNA methylation analysis

Genomic DNA was extracted from peripheral blood samples using the QIAamp DNA Blood Midi Kit (Qiagen, Hilden, Germany), followed by bisulfite conversion using the EZ DNA Methylation Kit (ZymoResearch, Irvine, CA, USA), hybridization using the Illumina Infinium HumanMethylation450 BeadChip (Illumina, San Diego, CA, USA), and scanning with the iScan System (Illumina). DNA methylation β-values, the ratio of methylation ranging from 0 (unmethylated) to 1 (methylated), were determined and normalized using the GenomeStudio Software (Illumina). None of the participants presented with > 1% of probes with a detection *p*-value > 0.01. Methylation probes with a detection *p*-value > 0.01 were removed if they were present in > 5% of the participants.

### Calculation of DNAm-based epigenetic age, TL, and blood cell composition

We used an online calculator (https://horvath.genetics.ucla.edu/html/dnamage/)^16^ to calculate DNAm-based epigenetic age, TL, and blood cell composition. We calculate DNAm age using Horvath’s multi-tissue clock^16^, Hannum’s single-tissue clock^17^, and Lu’s GrimAge clock^21^. EAA is the residual from regressing DNAm age on chronological age. A positive/negative EAA indicates that epigenetic age is higher/lower than expected from chronological age. We also investigated IEAA and EEAA. Further, we assessed the DNAmTL^22^. The age-adjusted DNAmTL (DNAmTLadjAge) is the residual from regressing DNAmTL on chronological age. A negative/positive DNAmTLadjAge indicates a shorter/longer than expected DNAmTL based on chronological age. Moreover, to assess DNAm-based blood cell composition, we determined the proportion of cytotoxic CD8+ T cells, helper CD4+ T cells, natural killer (NK) cells, B cells, monocytes, and granulocytes using Houseman’s method^52^, as well as the abundance of naive CD8+ T cells, exhausted CD8+ T cells, naive CD4+ T cells, and plasmablasts using Horvath’s method^16^.

### Statistics

Statistical analyses were performed using R version 3.5.2 (The R Foundation for Statistical Computing, Vienna, Austria) and EZR version 1.40 (Jichi Medical University, Saitama, Japan)^53^, which is a modified version of R commander. Between-group difference in the continuous variables was analyzed using a Student’s *t*-test, Welch’s *t*-test, or Mann–Whitney *U*-test, after testing for normal distribution and equality of variance. Relationship between continuous variables was analyzed using Pearson’s correlation coefficient or Spearman’s rank correlation coefficient, as appropriate. A multiple linear regression analysis was used to adjust for confounding factors, including sex, age, phenotype, and mood stabilizer medication status. Dummy variables were used where necessary. Statistical significance was defined as two-tailed *p* < 0.05.

## Results

**Fig. 1:**
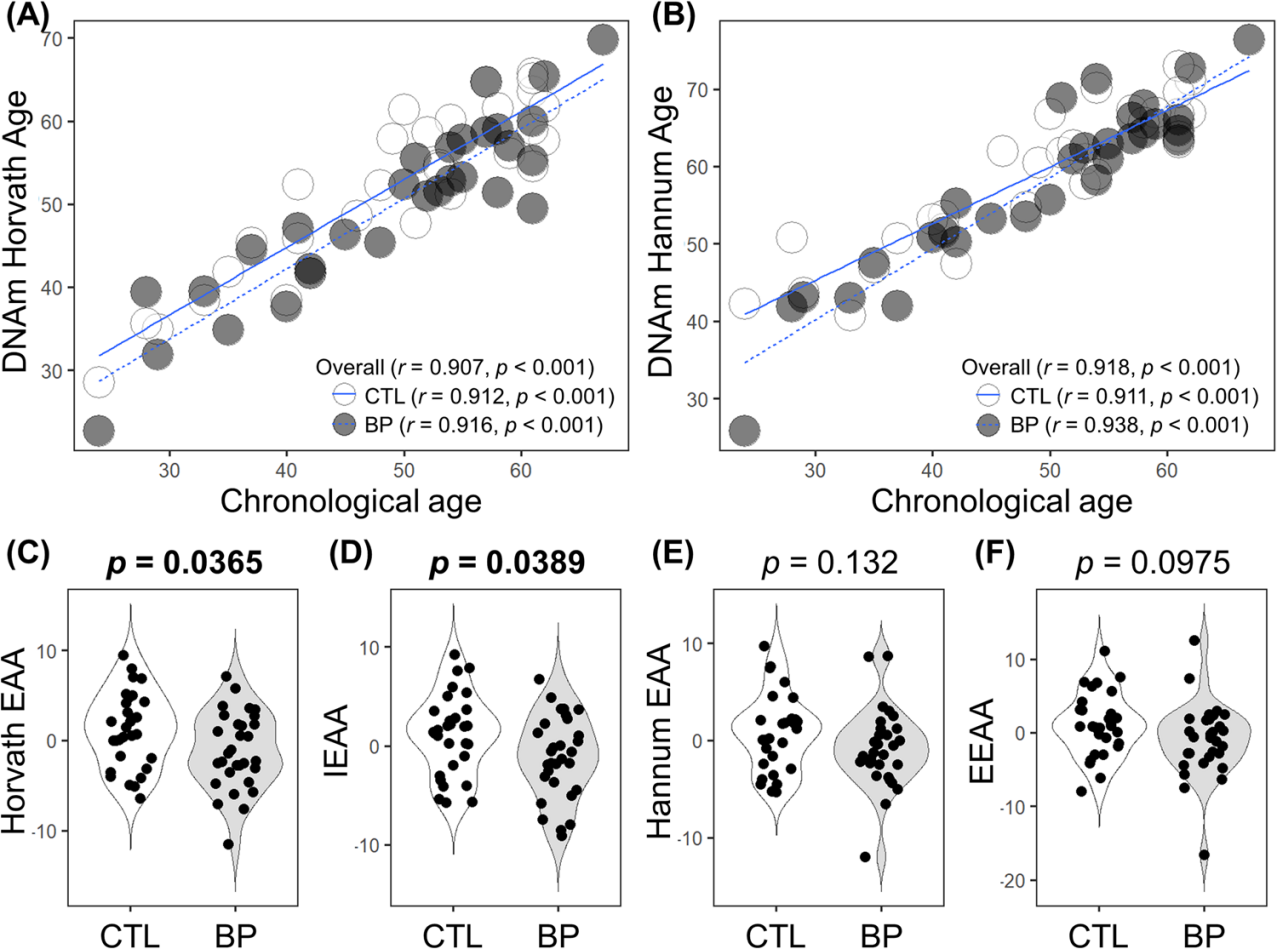
Horvath and Hannum epigenetic age acceleration. **a**, **b** Scatterplots show Horvath or Hannum Age vs. chronological age. Pearson’s correlation analysis indicated a significant correlation between DNA methylation age and chronological age in both groups. **c**–**f** Violin-plot with dots shows Horvath EAA, IEAA, Hannum EAA, or EEAA. Between-group comparisons were conducted using a Student’s *t*-test. Compared with controls, Horvath EAA (*p* = 0.0365) and IEAA (*p* = 0.0389) were significantly decreased in patients with BD. CTL, control; BD, bipolar disorder; EAA, epigenetic age acceleration; IEAA, intrinsic epigenetic age acceleration; EEAA, extrinsic epigenetic age acceleration.

**Fig. 2:**
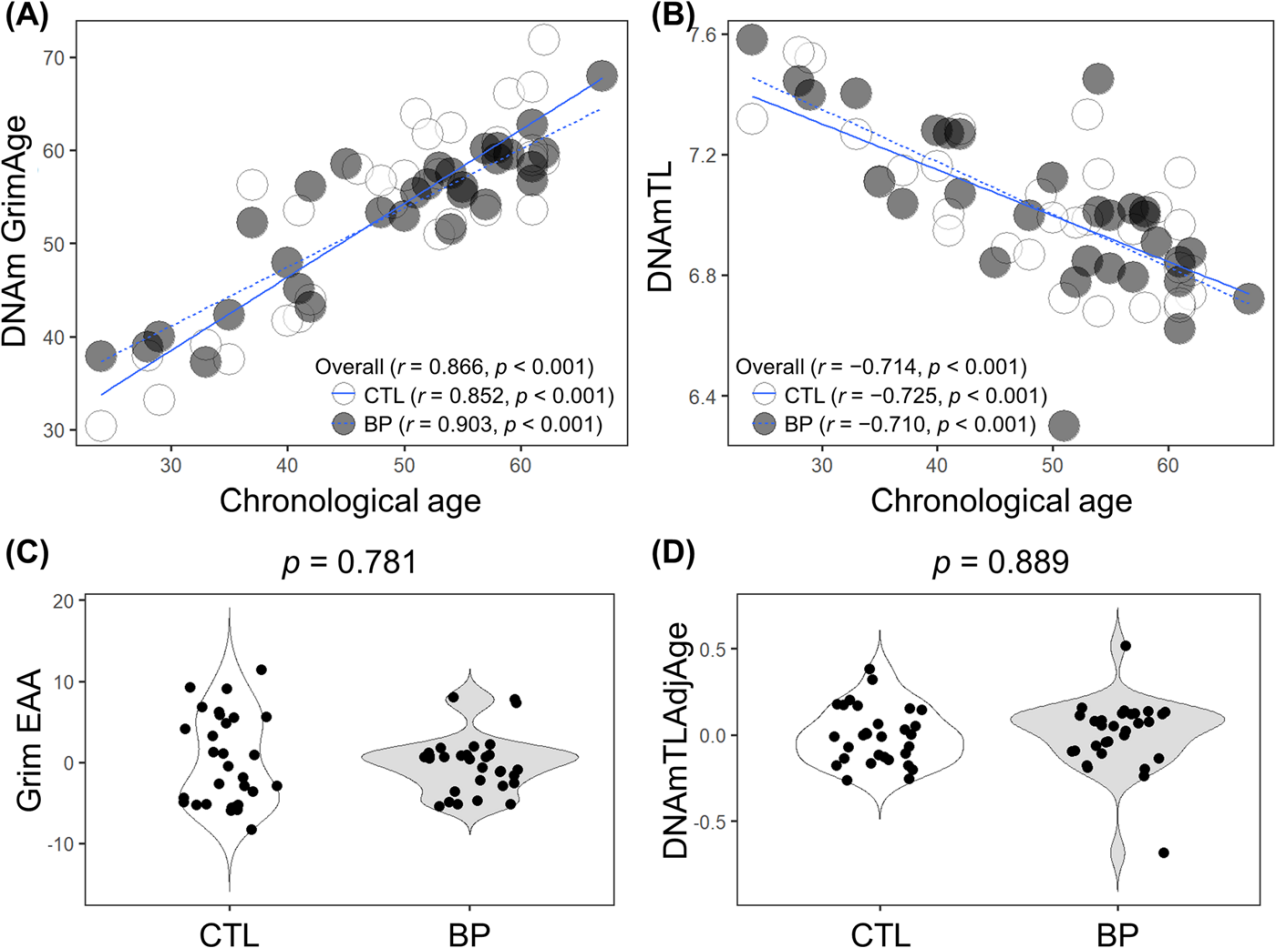
Grim epigenetic age acceleration and DNA methylation-based telomere length. **a**, **b** Scatterplots show GrimAge or DNAmTL vs. chronological age. Pearson’s correlation analysis indicated a significant correlation between GrimAge/DNAmTL and chronological age in both groups. **c**, **d** Violin-plot with dots shows Grim EAA or DNAmTLAdjAge. Welch’s *t*-test (Grim EAA) or Student’s *t*-test (DNAmTLAdjAge) showed no significant between-group differences. CTL, control; BD, bipolar disorder; EAA, epigenetic age acceleration; DNAmTL, DNA methylation-based telomere length; DNAmTLAdjAge, age-adjusted DNAmTL.

There was a significant correlation between chronological age and Horvath Age (*r* = 0.907, *p* < 0.001), Hannum Age (*r* = 0.918, *p* < 0.001), GrimAge (*r* = 0.866, *p* < 0.001), and DNAmTL (*r* = −0.714, *p* < 0.001) (Figs. 1 and 2). The strong linear relationships between each DNAm age/DNAmTL and chronological age indicated a valid high accuracy of the epigenetic estimator used in this study.

There was a significant between-group difference in Horvath EAA (*p* = 0.0364) and IEAA (*p* = 0.0389) but not in Hannum EAA (*p* = 0.132), EEAA (*p* = 0.0975), Grim EAA (*p* = 0.781), and DNAmTLAdjAge (*p* = 0.889) (Figs. 1, 2, and Table 1). After adjustment for confounding factors, including age and sex, there was a significant between-group difference in Horvath EAA (*p* = 0.0380) and IEAA (*p* = 0.0420) (Table 2). The between-group mean differences were 2.332 years for Horvath EAA and 2.232 years for IEAA, indicating that patients with BD were approximately two years younger than the controls. Moreover, subgroup analysis indicated no significant difference between patients with BD type I and II in the EAA measures or DNAmTLAdjAge (Supplementary Fig. S1).

**Table 2:** Multiple-linear regression analysis of DNA methylation-based epigenetic age acceleration and age-adjusted telomere length.

	Explanatory variable								
	Phenotype			Sex			Age		
Response variable	B	SE	*P*-value	B	SE	*P*-value	B	SE	*P*-value
*Model 1: phenotype-sex-age model in our study (n* *=* *60)*									
Horvath EAA	−2.331	1.097	**0.0380**	−1.164	1.103	0.296	0.005	0.049	0.916
IEAA	−2.233	1.073	**0.0420**	−0.538	1.079	0.620	0.003	0.048	0.952
Hannum EAA	−1.574	0.999	0.121	−2.333	1.005	**0.0239**	0.009	0.045	0.837
EEAA	−2.033	1.193	0.0940	−2.152	1.200	0.0783	0.009	0.053	0.871
Grim EAA	−0.340	1.143	0.767	−3.374	1.150	**0.00483**	0.013	0.051	0.807
DNAmTLAdjAge	0.007	0.048	0.891	−0.008	0.048	0.865	0.000	0.002	0.990

	Explanatory variable								
	Mood stabilizer medication status			Sex			Age		
Response variable	B	SE	*P*-value	B	SE	*P*-value	B	SE	*P*-value
*Model 2: mood stabilizer medication status-sex-age model in patients with bipolar disorder (n* *=* *30)*									
Horvath EAA	−4.050	1.713	**0.0261**	0.805	1.597	0.619	0.018	0.066	0.790
IEAA	−4.624	1.606	**0.00805**	1.772	1.497	0.248	0.013	0.062	0.841
Hannum EAA	−2.793	1.624	0.0978	−0.594	1.515	0.698	0.099	0.063	0.129
EEAA	−3.462	2.062	0.106	−0.086	1.923	0.965	0.110	0.080	0.180
Grim EAA	−1.739	1.454	0.243	−1.389	1.356	0.315	−0.070	0.056	0.225
DNAmTLAdjAge	0.135	0.085	0.126	−0.488	0.079	0.544	−0.001	0.003	0.731

*EAA* epigenetic age acceleration, *IEAA* intrinsic epigenetic age acceleration, *EEAA* extrinsic epigenetic age acceleration, *DNAmTLAdjAge* age-adjusted DNA methylation-based telomere length, *B* unstandardized partial regression coefficient, *SE* standard error.

Multiple linear regression analysis was performed with epigenetic age acceleration as the response variable and phenotype (model 1), mood stabilizer medication (lithium carbonate, sodium valproate, and carbamazepine) (model 2), sex, and age as the explanatory variables. Dummy variables were used as follows: phenotype, control = 0, bipolar disorder = 1; mood stabilizer medication status, none/mono medication = 0, combination medication = 1; sex, male = 0 and female = 1. Boldface type indicates significance.

Next, we investigated the effects of psychotropic medication on EAA in patients with BD. We observed a significant negative correlation between the CBZ medication dose and the four EAA measures (Horvath EAA, IEAA, Hannum EAA, and EEAA) (Supplementary Fig. S2). These results were consistent with the second analysis showing a significant association between the four EAA measures and CBZ medication status (used or non-used) (Supplementary Fig. S3).

**Fig. 3:**
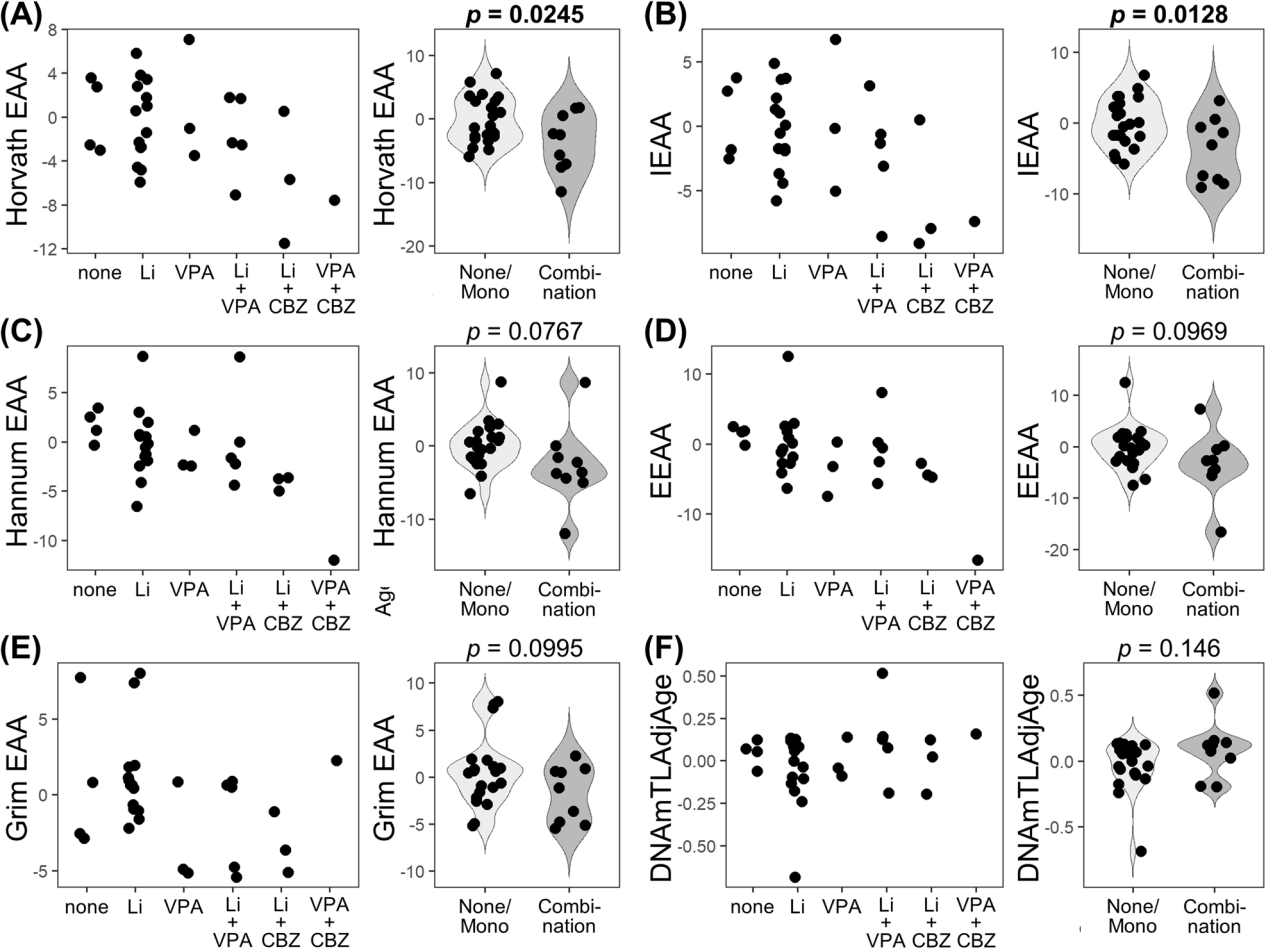
Epigenetic age acceleration and DNAmTLAdjAge vs. mood stabilizer medication in patients with bipolar disorder. Dot-plots and violin-plots with dots show mood stabilizers medication vs. **a** Horvath EAA, **b** IEAA, **c** Hannum EAA, **d** EEAA, **e** Grim EAA, or **f** DNAmTLAdjAge. Student’s *t*-test (Horvath EAA, IEAA, Hannum EAA, EEAA and DNAmTLAdjAge) or Welch’s *t*-test (Grim EAA) was performed for comparisons between patients with BD on combination medication and those on none/monotherapy of mood stabilizers (Li, VPA, or CBZ). Horvath EAA (*p* = 0.0245) and IEAA (*p* = 0.0128) were significantly decreased in patients with BD on combination medication. BD, bipolar disorder; EAA, epigenetic age acceleration; IEAA, intrinsic epigenetic age acceleration; EEAA, extrinsic epigenetic age acceleration; DNAmTLAdjAge, age-adjusted DNA methylation-based telomere length; Li, lithium carbonate; VPA, sodium valproate; CBZ, carbamazepine.

These findings indicate that CBZ decreased EAA. However, all the patients with BD using CBZ were under a combination of medications with Li or VPA (Fig. 3). Therefore, we investigated the effects of the combination medications and observed a significant difference in Horvath EAA (*p* = 0.0245) and IEAA (*p* = 0.0128) between patients with BD using and not using combination medications (Fig. 3). After adjustment for confounding factors, including age and sex, the significant difference in the Horvath EAA (*p* = 0.0261) and IEAA (*p* = 0.00805) remained (Table 2). Moreover, we adjusted for the age of onset or duration of illness. Given their correlation with age (*r* = 0.466, *p* = 0.00950 and *r* = 0.531, *p* = 0.00254, respectively), we replaced them with age considering multicollinearity. The significant difference in the Horvath EAA and IEAA remained (Supplementary Table S1).

On the other hand, there was no correlation of Grim EAA with mood stabilizer dose; however, it was positively correlated with antipsychotics (Supplementary Fig. S4). There was no correlation of DNAmTLAdjAge with any psychotropic medication (Supplementary Fig. S4).

Further, we investigated between-group differences in the leukocyte cell composition. We found that patients with BD had fewer CD8+ T cells (*p* = 0.0306) (Supplementary Fig. S5). After adjustment for confounding factors, including age and sex, the significant difference in the CD8+ T cell population remained (*p* = 0.0166) (Supplementary Table S2).

## Discussion

We investigated EAA measures, including Horvath EAA, IEAA, Hannum EAA, EEAA, and Grim EAA, DNAmTL, and DNAm-based blood cell composition in patients with BD and healthy controls. There was a significant decrease in Horvath EAA and IEAA in patients with BD. Furthermore, the decreased Horvath EAA and IEAA were associated with combination medications of the three mood stabilizers (Li, VPA, and CBZ). Moreover, there were significantly fewer CD8+ T cells in patients with BD compared with that in controls.

These findings are inconsistent with those of a previous study^45^, which reported increased Horvath EAA in the blood of older (>33 years old), but not younger, patients with BD. However, our participants had a higher usage rate of mood stabilizers (Li, 73.3%; VPA, 30.0%; CBZ, 13.3%) than those in the previous study (Li, 23.8%; anticonvulsants, 23.8%). Moreover, there was an age difference between the participants in our study (controls: mean age ± SD, 48.8 ± 11.3; patients with BD, 49.0 ± 11.4) and those in the previous study (controls, 34.75 ± 10.0; patients with BD, 33.95 ± 9.3). Principally, EAA differences are more likely to be observed among elderly individuals. These findings suggest that BD increases the EAA while the long-term use of mood stabilizers, including Li, VPA, and CBZ, could repress EAA. Li, which is a first-line mood stabilizer, has been shown to repress suicide^54^, which contributes to the high mortality in patients with BD^2^. Therefore, we presumed that the high usage rate of mood stabilizers in this study contributed to the observed decreased EAA.

TL is the most common biological aging estimator; however, there have been inconsistent reports on the TL in patients with BD^5–14^, including the presently studied DNAmTL. This could be attributed to the association between long-term Li medication and longer telomeres in patients with BD independent of the response to therapy, and the fact that Li treatment increases the expression of telomerase gene, which counteracts telomere shortening, in human neural progenitor cells^13,14^. Moreover, a previous study revealed that three mood stabilizers (Li, VPA, and CBZ) have common epigenetic targets and a predisposition to alter DNAm levels by genome-wide DNAm analysis on approximately 27,000 CpG sites using human neuroblastoma cells treated with the mood stabilizers^43^. There is a need for further studies on the association between mood stabilizers and EAA.

We found decreased IEAA, but not EEAA, in patients with BD. IEAA indicates cell-intrinsic aging independent of age-related changes in blood cell composition^19,20^. Both IEAA and EEAA are correlated with metabolic syndrome; further, EEAA is more strongly correlated with lifestyle factors, including diet, education, income, and body mass index (BMI) than IEAA^20^. Therefore, there is a need for further studies to elucidate the effects of lifestyle factors on epigenetic aging in patients with BD.

We previously reported decreased EEAA in hospitalized patients with SCZ^36^ and increased EEAA in suicide completers^31^. In contrast, other previous studies have reported increased Horvath EAA in patients with MDD^34^ and elderly individuals with BD^45^, as well as increased Hannum EAA in individuals with childhood trauma and PTSD severity^35^. Epigenetic clocks capture both chronological and biological information dependently upon the CpG sites used to develop the clock^55^. This indicates that the EAA changes associate with psychiatric disorders and/or psychotropic drug treatments in different manners. An additional in silico analysis using DAVID Bioinformatics Resources 6.8 (https://david.ncifcrf.gov/)^56,57^ on genes co-located with the Horvath EAA and IEAA 353 CpG sites showed enrichment not only for cancer similar to previous studies^58,59^, but also for “psychiatric disorders” (Supplementary Table S3). Further studies are required to elucidate the causal relationship between EAA and psychiatric disorders.

There was no correlation of Grim EAA with mood stabilizers, though it was positively correlated with antipsychotics. However, antipsychotics are second-line medications in BP treatment and used in case of severe symptoms. There is a need for careful evaluation of the potential causality.

Additionally, we observed significantly fewer CD8+ T cells in patients with BD, which is consistent with previous findings^48–50^. This demonstrates that the DNAm-based approach replicated the results of previous studies using flow cytometry. The classic role of CD8+ T cells involves host defense against intracellular infectious agents such as viruses. Indeed, patients with BD are more susceptible to infections such as hepatitis B/C and human immunodeficiency viruses^60,61^. Our findings of decreased CD8+ T cells validate the hypothesis of an association between BD and viral infections^48^. There is a need for further studies to elucidate the availability of T lymphocytes subpopulations as trait/state BD biomarkers. Moreover, our findings suggest the availability of DNAm-based surrogate measures of blood cell composition as biomarkers of psychiatric diseases, such as BD. This method allows for measurement of the blood cell composition using archival samples, which contrasts with flow cytometry as it requires fresh samples^52^.

This study has several limitations. First, the sample size of our cohort was relatively small. Previous studies indicate that BD type I and II might have genetically different clinical pictures^62^. Larger scale studies are needed to confirm our findings in each BD subtype. Second, there is a lack of information regarding potential confounders affecting EAA, including smoking, alcohol, BMI, occupation, medical conditions: disease phase ([hypo-] manic, remitted, and depressed), disease severity, and the number of hospitalizations/affective episodes. Indeed, gene expression differences are reported to be related to the disease stage^63^. Third, we could not obtain detailed information regarding the mood stabilizers, including their blood concentration and administration duration, the treatment outcome of combined and none/monotherapy treatment, and their association with EAA. Fourth, we only used blood samples; therefore, we cannot rule out the possibility of accelerated epigenetic aging in other tissues or defined cell types in patients with BD.

Although we observed an association of EAA with BD and mood stabilizers, further studies are required to determine the clinical implications of this. Investigations using postmortem brain samples and other tissues/cell types, including neural/glial cells, are required to develop more accurate tissue/cell-specific epigenetic clocks in the future. Moreover, approaches using human induced pluripotent stem cells will permit complete resetting of epigenetic age and targeting of distinct cells^16,64^. Combining these approaches, the understanding of the molecular mechanisms underlying BD and the effects of mood stabilizers on the epigenetic aging could be improved, which could lead to the prevention of BD and improved treatment.

In conclusion, this study provided novel evidence for decreased Horvath EAA and IEAA in patients with BD. Moreover, this is the first report on the potential effects of mood stabilizers (Li, VPA, and CBZ) on EAA repression in patients with BD. These findings might be relevant for the identification of the molecular mechanisms underlying these newly observed effects of mood stabilizers. Further studies on epigenetic aging could provide a promising new line of investigation for elucidating the molecular mechanisms of BD and mood stabilizers.

## Supplementary information

Supplementary Figure S1

Supplementary Figure S2

Supplementary Figure S3

Supplementary Figure S4

Supplementary Figure S5

Supplementary Figure Legends

Supplementary Table S1

Supplementary Table S2

Supplementary Table S3

## Data Availability

The data that support the findings of this study are available from the corresponding author upon reasonable request.
