# Supplementary figures and images for: Decelerated epigenetic aging associated with mood stabilizers in the blood of patients with bipolar disorder
Source: Transl Psychiatry. 2020 May 4;10:129. doi: 10.1038/s41398-020-0813-y (PMC7198548; doi:10.1038/s41398-020-0813-y)

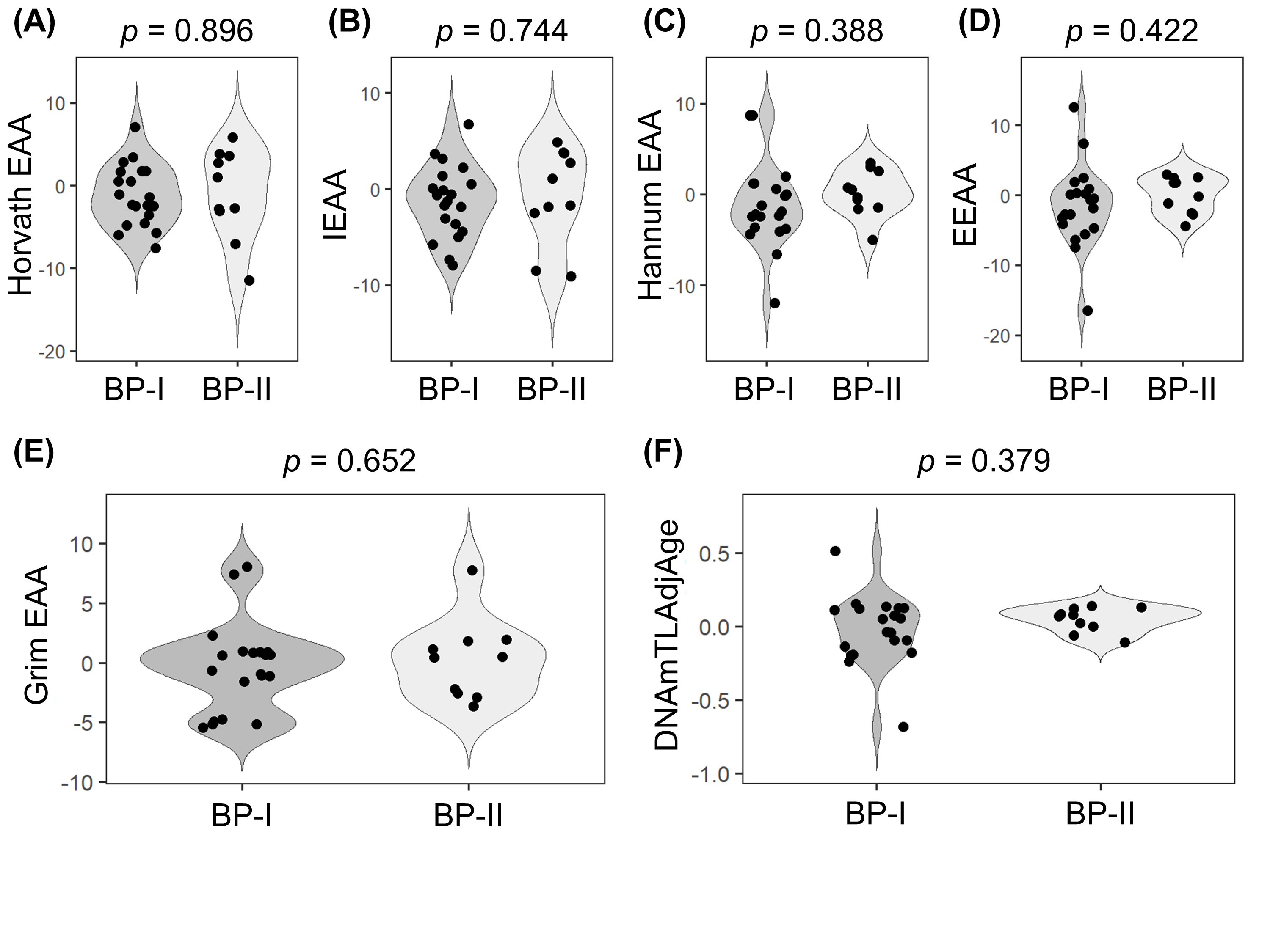

Supplement: Supplementary file 1 — Supplementary Figure S1 [file 41398_2020_813_MOESM1_ESM.tif]

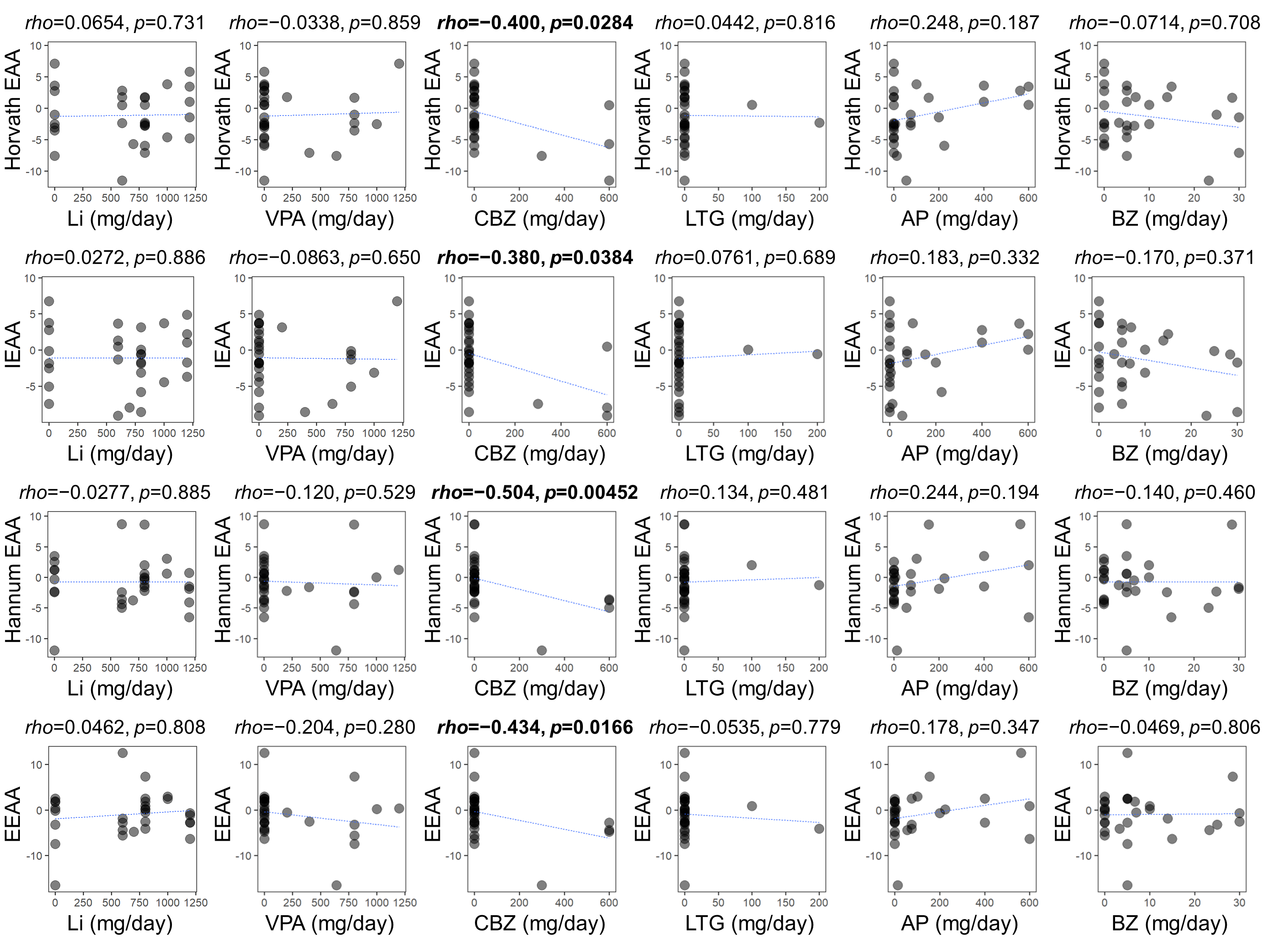

Supplement: Supplementary file 2 — Supplementary Figure S2 [file 41398_2020_813_MOESM2_ESM.tif]

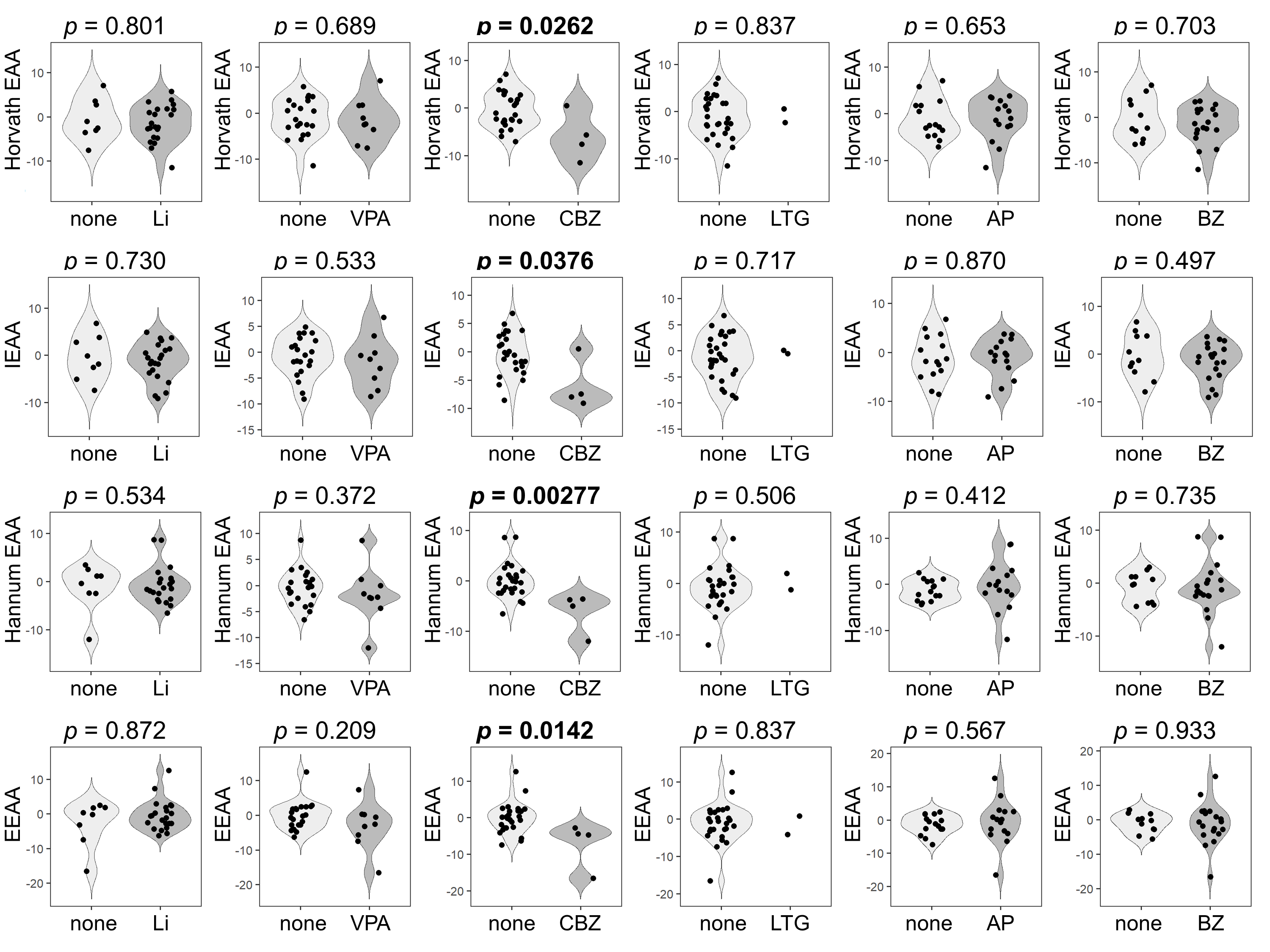

Supplement: Supplementary file 3 — Supplementary Figure S3 [file 41398_2020_813_MOESM3_ESM.tif]

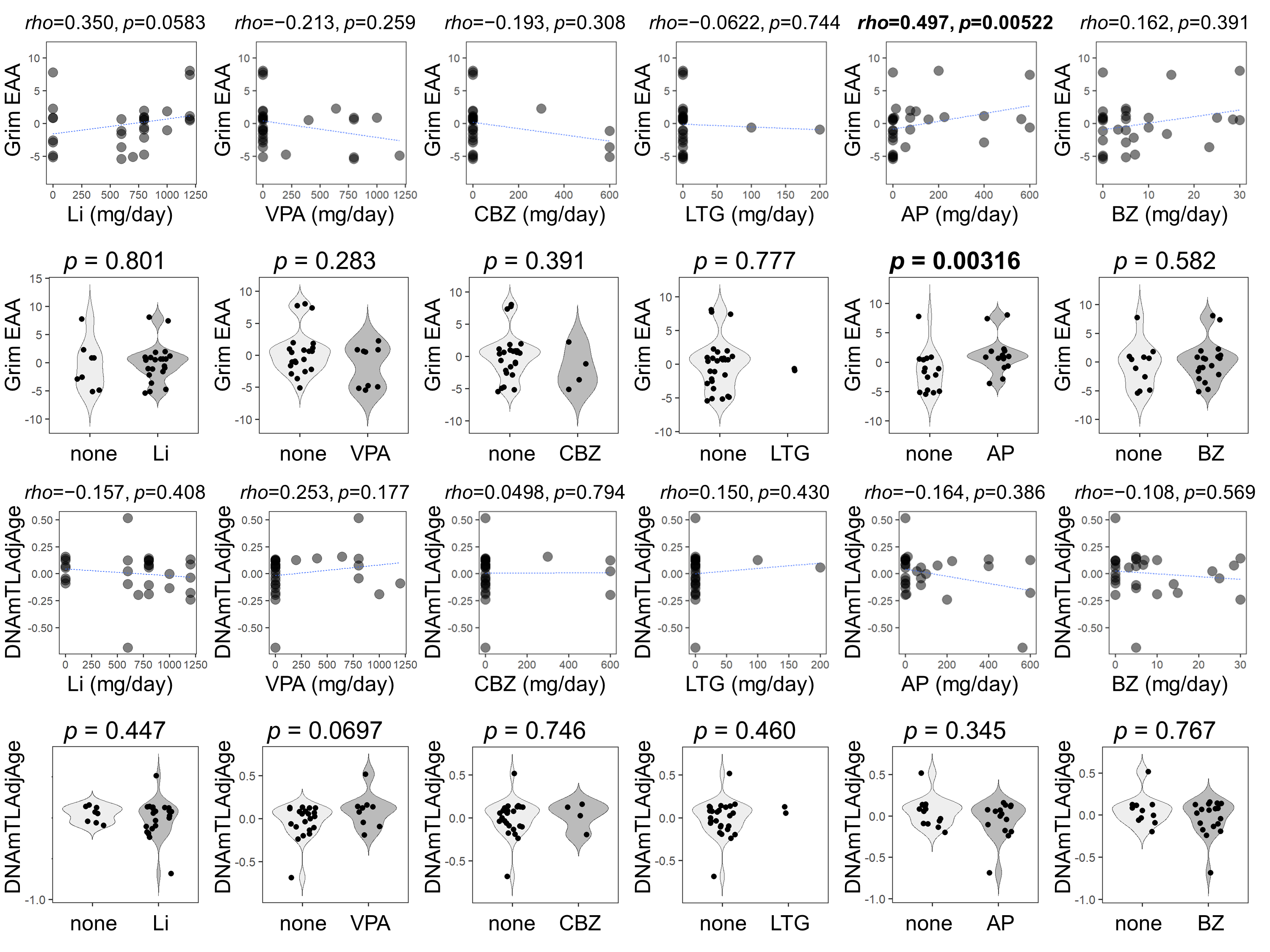

Supplement: Supplementary file 4 — Supplementary Figure S4 [file 41398_2020_813_MOESM4_ESM.tif]

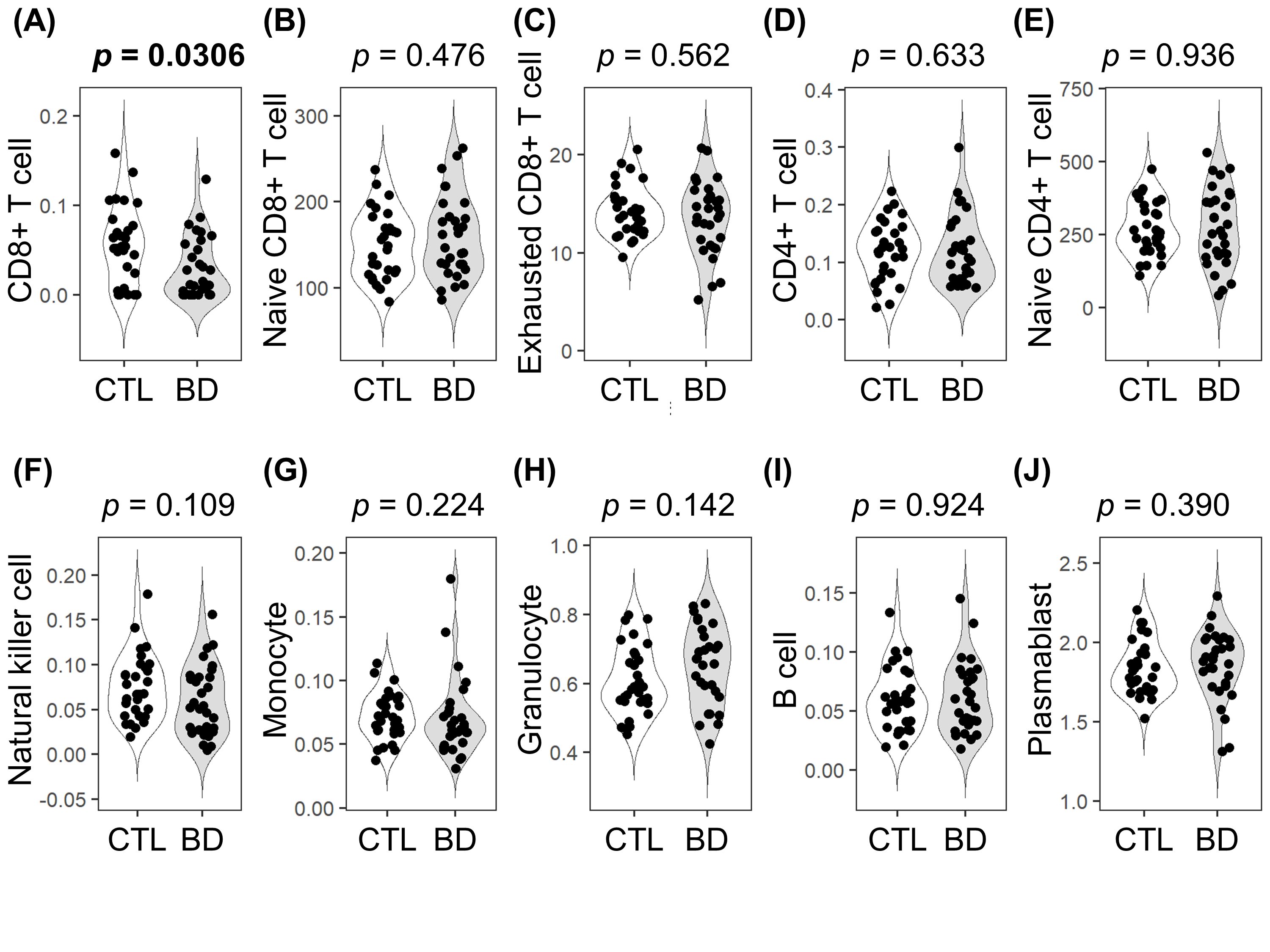

Supplement: Supplementary file 5 — Supplementary Figure S5 [file 41398_2020_813_MOESM5_ESM.tif]
