# Supplementary material for: Decelerated epigenetic aging associated with mood stabilizers in the blood of patients with bipolar disorder
Source: Transl Psychiatry. 2020 May 4;10:129. doi: 10.1038/s41398-020-0813-y (PMC7198548; doi:10.1038/s41398-020-0813-y)
Supplement: Supplementary file 6 — Supplementary Figure Legends [file 41398_2020_813_MOESM6_ESM.docx]

**Supplementary Figure legends**

**Supplementary Fig. S1. Epigenetic age acceleration between bipolar type I and II.** Violin-plots with dots show (**A**) Horvath EAA, (**B**) IEAA, (**C**) Hannum EAA, (**D**) EEAA, (**E**) Grim EAA, or (**F**) DNAmTLAdjAge. Student’s t-test (Horvath EAA, IEAA, Hannum EAA, EEAA, and DNAmTLAdjAge) or Welch’s t-test (Grim EAA) did not indicate a significant between-group difference.

BD-I, bipolar disorder type I; BD-II, bipolar disorder type II; EAA, epigenetic age acceleration; IEAA, intrinsic epigenetic age acceleration; EEAA, extrinsic epigenetic age acceleration; DNAmTLAdjAge, age-adjusted DNA methylation-based telomere length.

**Supplementary Fig. S2. Epigenetic age acceleration vs. psychotropic drug doses.** Scatterplots show Horvath EAA, IEAA, Hannum EAA, or EEAA vs. dose of psychotropic drugs. The AP and BZ doses were calculated as chlorpromazine and diazepam equivalents, respectively. Spearman’s correlation analysis indicates that Horvath EAA, IEAA, Hannum EAA, and EEAA were significantly negatively correlated with the CBZ dose.

EAA, epigenetic age acceleration; IEAA, intrinsic epigenetic age acceleration; EEAA, extrinsic epigenetic age acceleration; Li, lithium carbonate; VPA, sodium valproate; CBZ carbamazepine; LTG, lamotrigine; AP, antipsychotic; BZ, benzodiazepine.

**Supplementary Fig. S3. Epigenetic age acceleration vs. status of psychotropic drugs.** Violin-plots with dots show Horvath EAA, IEAA, Hannum EAA, or EEAA vs. status of psychotropic drugs. Mann-Whitney U-test was performed for comparisons between patients with BD using and not using each psychotropic drug. Horvath EAA, IEAA, Hannum EAA, and EEAA were significantly decreased in patients with BD on CBZ medication.

EAA, epigenetic age acceleration; IEAA, intrinsic epigenetic age acceleration; EEAA, extrinsic epigenetic age acceleration; Li, lithium carbonate; VPA, sodium valproate; CBZ carbamazepine; LTG, lamotrigine; AP, antipsychotic; BZ, benzodiazepine.

**Supplementary Fig. S4. Grim EAA/DNAmTLAdjAge vs. dose/status of psychotropic drugs.** Violin-plots with dots show Grim EAA or DNAmTLAdjAge vs. dose/status of psychotropic drugs. Spearman’s correlation analysis showed a positive correlation of Grim EAA with the AP dose. Mann-Whitney U-test indicated a significant increase in Grim EAA in patients with BD on AP medication.

EAA, epigenetic age acceleration; DNAmTLAdjAge, age-adjusted DNA methylation-based telomere length; Li, lithium carbonate; VPA, sodium valproate; CBZ carbamazepine; LTG, lamotrigine; AP, antipsychotic; BZ, benzodiazepine.

**Supplementary Fig. S5. Blood cell counts vs. bipolar disorder.** Violin-plots with dots show BD status (x-axis) vs. (**A**) cytotoxic CD8+ T cells, (**B**) naive CD8+ T cells, (**C**) exhausted CD8+ T cells (both memory and effector T cells), (**D**) helper CD4+ T cells, (**E**) naive CD4+ T cells, (**F**) natural killer cells, (**G**) monocytes, (**H**) granulocytes, (**I**) B cells, and (**J**) plasmablasts. The abundance of blood cell counts was estimated based on DNA methylation levels using the online epigenetic clock software. The y-axis in (**A**, **D**, **F**, **G**, **H**, and **I**) reports the estimated proportions based on Houseman’s method[^52^](#_ENREF_52) while that in (**B**, **C**, **E**, and **J**) presents ordinal abundance measures based on Horvath’s method.[^16^](#_ENREF_16) Between-group comparisons using the Mann-Whitney U-test showed a higher CD8+ T cell percentage (*p* = 0.0306) in patients with BD.

CTL, control; BD, bipolar disorder.
