# Supplementary material for: Decelerated epigenetic aging associated with mood stabilizers in the blood of patients with bipolar disorder
Source: Transl Psychiatry. 2020 May 4;10:129. doi: 10.1038/s41398-020-0813-y (PMC7198548; doi:10.1038/s41398-020-0813-y)
Supplement: Supplementary file 7 — Supplementary Table S1 [file 41398_2020_813_MOESM7_ESM.docx]

**Supplementary Table S1. Multiple-linear regression analysis of DNA methylation-based epigenetic age acceleration and age-adjusted telomere length using the age of onset/duration of illness in patients with bipolar disorder (n = 30).**

| **Model 1: mood stabilizer medication status-sex-age of onset model** | | | | | | | | | | | | |
| --- | --- | --- | --- | --- | --- | --- | --- | --- | --- | --- | --- | --- |
|  | | Explanatory variable | | | | | | | | | | |
|  | Mood stabilizer medication status | | | |  | Sex |  |  |  | Age of onset | | |
| Response variable | B | | SE | *P*-value |  | B | SE | *P*-value |  | B | SE | *P*-value |
| Horvath EAA | −3.877 | | 1.815 | **0.0426** | | 0.697 | 1.671 | 0.680 | | 0.020 | 0.074 | 0.787 |
| IEAA | −4.510 | | 1.702 | **0.0137** | | 1.702 | 1.567 | 0.288 | | 0.013 | 0.070 | 0.849 |
| Hannum EAA | −1.104 | | 1.505 | 0.470 | | −1.810 | 1.386 | 0.203 | | 0.204 | 0.062 | **0.00287** |
| EEAA | −1.526 | | 1.959 | 0.443 | | −1.487 | 1.807 | 0.417 | | 0.234 | 0.080 | **0.00747** |
| Grim EAA | −2.002 | | 1.582 | 0.217 | | −1.313 | 1.457 | 0.376 | | −0.029 | 0.065 | 0.660 |
| DNAmTLAdjAge | 0.134 | | 0.090 | 0.150 | | −0.051 | 0.083 | 0.547 | | 0.000 | 0.004 | 0.998 |
| **Model 2: mood stabilizer medication status-sex-duration of illness model** | | | | | | | | | | | | |
|  | Explanatory variable | | | | | | | | | | | |
|  | Mood stabilizer medication status | | | |  | Sex |  |  |  | Duration of illness | | |
| Response variable | B | | SE | *P*-value |  | B | SE | *P*-value |  | B | SE | *P*-value |
| Horvath EAA | −4.121 | | 1.812 | **0.0318** | | 0.883 | 1.634 | 0.594 | | 0.009 | 0.069 | 0.894 |
| IEAA | −4.699 | | 1.698 | **0.0105** | | 1.842 | 1.531 | 0.240 | | 0.009 | 0.065 | 0.885 |
| Hannum EAA | −2.168 | | 1.763 | 0.230 | | −0.779 | 1.590 | 0.628 | | −0.068 | 0.067 | 0.318 |
| EEAA | −2.718 | | 2.220 | 0.232 | | −0.321 | 2.001 | 0.874 | | −0.082 | 0.084 | 0.342 |
| Grim EAA | −1.348 | | 1.563 | 0.396 | | −1.763 | 1.409 | 0.222 | | −0.049 | 0.059 | 0.414 |
| DNAmTLAdjAge | 0.144 | | 0.090 | 0.121 | | −0.057 | 0.081 | 0.491 | | −0.001 | 0.003 | 0.737 |

EAA, epigenetic age acceleration; IEAA, intrinsic epigenetic age acceleration; EEAA, extrinsic epigenetic age acceleration; DNAmTLAdjAge, age-adjusted DNA methylation-based telomere length; B, unstandardized partial regression coefficient; SE, standard error.

Multiple linear regression analysis was performed with epigenetic age acceleration as the response variable and mood stabilizer medication (lithium carbonate, sodium valproate, and carbamazepine), sex, age of onset (model 1), and duration of illness (model 2) as the explanatory variables. Dummy variables were used as follows: phenotype, control = 0, bipolar disorder = 1; mood stabilizer medication status, none/mono medication = 0, combination medication = 1; sex, male = 0 and female = 1. Boldface type indicates significance.
