# Supplementary material for: Decelerated epigenetic aging associated with mood stabilizers in the blood of patients with bipolar disorder
Source: Transl Psychiatry. 2020 May 4;10:129. doi: 10.1038/s41398-020-0813-y (PMC7198548; doi:10.1038/s41398-020-0813-y)
Supplement: Supplementary file 8 — Supplementary Table S2 [file 41398_2020_813_MOESM8_ESM.docx]

**Supplementary Table S2. Multiple-linear regression analysis of DNA methylation-based blood cell composition in our study (n = 60).**

|  | Explanatory variable | |  | |  |  |  | |  |  |  |
| --- | --- | --- | --- | --- | --- | --- | --- | --- | --- | --- | --- |
|  | Phenotype |  |  |  | Sex |  |  |  | Age |  |  |
| Response variable | B | SE | *P*-value | | B | SE | *P*-value | | B | SE | *P*-value |
| CD8+ T cell | −0.024 | 0.010 | **0.0166** | | 0.015 | 0.010 | 0.126 | | −0.001 | 0.000 | 0.127 |
| Naive CD8+ T cell | 9.026 | 10.176 | 0.379 | | 9.245 | 10.233 | 0.370 | | −1.635 | 0.456 | **0.000711** |
| Exhausted CD8+ T cell | −0.784 | 0.807 | 0.336 | | 1.320 | 0.812 | 0.109 | | 0.066 | 0.036 | 0.0739 |
| CD4+ T cell | −0.001 | 0.014 | 0.953 | | 0.013 | 0.014 | 0.371 | | 0.001 | 0.001 | 0.0758 |
| Naive CD4+ T cell | 2.596 | 28.886 | 0.929 | | 53.370 | 29.049 | 0.0715 | | −0.012 | 1.295 | 0.993 |
| Natural killer cell | −0.015 | 0.010 | 0.132 | | 0.003 | 0.010 | 0.756 | | 0.000 | 0.000 | 0.304 |
| Monocyte | −0.002 | 0.007 | 0.776 | | 0.003 | 0.007 | 0.678 | | 0.000 | 0.000 | 0.497 |
| Granulocyte | 0.039 | 0.027 | 0.152 | | −0.048 | 0.027 | 0.0774 | | −0.002 | 0.001 | 0.142 |
| B cell | 0.000 | 0.007 | 0.948 | | 0.0182 | 0.007 | **0.0124** | | 0.000 | 0.000 | 0.163 |
| Plasmablast | 0.021 | 0.049 | 0.678 | | 0.048 | 0.050 | 0.341 | | −0.005 | 0.002 | **0.0301** |

B, unstandardized partial regression coefficient; SE, standard error.

Multiple linear regression analysis was performed with epigenetic age acceleration as the response variable and phenotype, sex, and age as the explanatory variables. Dummy variables were used as follows: phenotype, control = 0, bipolar disorder = 1; sex, male = 0 and female = 1. Boldface type indicates significance.
