# Supplementary material for: Decelerated epigenetic aging associated with mood stabilizers in the blood of patients with bipolar disorder
Source: Transl Psychiatry. 2020 May 4;10:129. doi: 10.1038/s41398-020-0813-y (PMC7198548; doi:10.1038/s41398-020-0813-y)
Supplement: Supplementary file 9 — Supplementary Table S3 [file 41398_2020_813_MOESM9_ESM.docx]

**Supplementary Table S3. Pathway analysis of 353 CpG sites of Horvath’s clock.**

| Term | Count | % | *P*-value | Benjamini FDR |
| --- | --- | --- | --- | --- |
| **GAD_DISEASE** |  |  |  |  |
| Leukemia, Lymphocytic, Chronic, B-Cell | 13 | 3.8 | 0.00049 | 0.33 |
| Tobacco Use Disorder | 75 | 21.8 | 0.0026 | 0.65 |
| Psychiatric Disorders | 6 | 1.7 | 0.011 | 0.95 |
| prostate cancer | 17 | 4.9 | 0.023 | 0.99 |
| Vascular Diseases | 3 | 0.9 | 0.029 | 0.99 |
| Type 2 Diabetes\| edema \| rosiglitazone | 51 | 14.8 | 0.048 | 1.00 |
| Bulimia | 8 | 2.3 | 0.049 | 1.00 |
| gamma-Glutamylcyclotransferase | 3 | 0.9 | 0.064 | 1.00 |
| stroke, ischemic | 4 | 1.2 | 0.084 | 1.00 |
| alcohol consumption | 6 | 1.7 | 0.087 | 1.00 |
| **KEGG_PATHWAY** |  |  |  |  |
| Pathways in cancer | 16 | 4.7 | 0.0036 | 0.53 |
| Gap junction | 7 | 2 | 0.0044 | 0.37 |
| Small cell lung cancer | 6 | 1.7 | 0.017 | 0.69 |
| Non-alcoholic fatty liver disease (NAFLD) | 8 | 2.3 | 0.017 | 0.59 |
| Long-term depression | 5 | 1.5 | 0.021 | 0.59 |
| Melanoma | 5 | 1.5 | 0.036 | 0.72 |
| Non-small cell lung cancer | 4 | 1.2 | 0.075 | 0.91 |
| Regulation of lipolysis in adipocytes | 4 | 1.2 | 0.075 | 0.91 |
| Rap1 signaling pathway | 8 | 2.3 | 0.076 | 0.88 |
| Circadian entrainment | 5 | 1.5 | 0.086 | 0.88 |
| FoxO signaling pathway | 6 | 1.7 | 0.087 | 0.85 |

Analysis of DAVID Bioinformatics Resources 6.8 (https://david.ncifcrf.gov/) was performed for the genes co-located with the Horvath 353 CpG sites.
